# Use of repetitive transcranial magnetic stimulation in an adolescent with autism spectrum disorder and comorbid major depression disorder with anxiety symptoms: A case study

**DOI:** 10.1192/j.eurpsy.2021.607

**Published:** 2021-08-13

**Authors:** M. Nystazaki, S. Kalimeris, A. Tsakiri, E. Tsalamanios, P. Gkikas

**Affiliations:** 1 Psychiatric And Neurological Center, SMART CNS CENTER, ATHENS, Greece; 2 Child And Adolescent Psychiatric Clinic, Asklipieio General Hospital of Athens, Athens, Greece

**Keywords:** r-TMS, major depression disorder, autism spectrum disorder

## Abstract

**Introduction:**

Psychiatric comorbidities, including depressive and anxiety disorders, are common in individuals with autism spectrum disorder (ASD). Use of conventional therapies for treating depression and anxiety are of limited efficacy in individuals with ASD making treatment a challenging field. Repetitive Transcranial Magnetic Stimulation (rTMS) is a safe and efficacious technique in major depressive disorder, and a similar approach could yield therapeutic benefits in ASD.

**Objectives:**

The aim of this case study is to present the effectiveness of rTMS in a 17 year old patient diagnosed with ASD and comorbid major depression disorder with anxiety symptoms.

**Methods:**

This is a case study of a male adolescent aged 17, diagnosed with ASD and comorbid major depression disorder with anxiety symptoms, suicidal ideation and aggressive behavior. The protocol applied was 4 weeks of daily rTMS sessions. This involved rTMS to the left dorsomedial prefrontal cortex (10 Hz, 3.000 pulses/120% motor threshold) to treat depressive symptoms and to the right (50Hz, 600 pulses/ 120%motor threshold) to treat anxiety symptoms. Assessments were conducted using the BDI, PHQ-9 and GAD-7 scales at baseline and one month follow up. Suicidal ideation and aggressive behavior were assessed by a clinician at same intervals.

**Results:**

Patient showed overall improvement in scores both in depression and anxiety scales. Suicidal Ideation and aggressive behavior showed significant reduction. No side effects were recorded during therapy.

**Conclusions:**

Our findings suggest that the use of rTMS therapy in adolescents in the autistic spectrum and comorbid major depression disorder and anxiety symptoms is an efficacious and safe therapeutic treatment option.

